# Autopsy diagnosis of leptomeningeal carcinomatosis, the first manifestation of gastric adenocarcinoma: a rare case report and review of the literature 

**Published:** 2021

**Authors:** Bita Geramizadeh, Mehran Fereidooni, Alireza Dehghan, Kamran Bagheri-Lankarani

**Affiliations:** 1 *Department of Pathology, Medical School of Shiraz University, Shiraz University of Medical Sciences, Shiraz, Iran*; 2 *Transplant Research Center, Shiraz University of Medical Sciences, Shiraz, Iran, Shiraz, Iran*; 3 *Legal Medicine research Center, Legal Medicine Organization, Tehran, Iran*; 4 *Department of Radiology, Shiraz University of Medical Sciences, Shiraz, Iran*; 5 *Health Policy Research Center, Institute of Health, Shiraz University of Medical Sciences, Shiraz, Iran *

**Keywords:** leptomeningeal carcinomatosis, gastric adenocarcinoma, autopsy

## Abstract

This study aimed to report an unusual presentation of an advanced gastric adenocarcinoma. Leptomeningeal carcinomatosis is a rare event in gastric adenocarcinoma. It is much more uncommon as the primary manifestation in post-mortem evaluation of the cause of death in a patient presenting with headache and neurological signs and symptoms. Herein, we discuss our experience with a case of gastric adenocarcinoma, who was diagnosed after death, presenting with neurological signs and symptoms of leptomeningeal carcinomatosis. A 52-year-old gentleman presented with intractable headache and neck pain as well as vertigo. His physical examination showed only decreased deep tendon reflexes. He died after a short period of coma. Post-mortem evaluation showed numerous signet ring cells in the subarachnoid space as well as gastric malignant ulcer. In patients with intractable headache with no identifiable cause, meningeal involvement and infiltration should be considered as the probable underlying cause. Radiologic findings are not significant; however, lumbar puncture can be diagnostic.

## Introduction

 Leptomeningeal carcinomatosis (LMC) is defined as the infiltration of the pia and arachnoid membrane by malignant epithelial cells (1). The reported incidence of LMC is 5%-8% in cancer patients; however, the clinical diagnosis of LMC has been identified in 2%-4% of patients who were found to have LMC on autopsy. This incidence depends on the type of carcinoma. Most reported cases have originated from the breast and lung. LMC from gastric adenocarcinoma is rare and has been reported in 0.16% of cases with gastric cancers, the majority of which has been part of disseminated disease (2,3). LMC as the first manifestation of gastric adenocarcinoma is extremely rare. To the best of our knowledge, there has been only 9 case reports in the English literature in the last 20 years (4-12). Herein, we report our experience with a patient who presented with headache and neck pain in whom the diagnosis of gastric adenocarcinoma was made after autopsy. 

## Case Report

A 52-year-old gentleman referred to his general practitioner (GP) with an intractable headache with a one-week duration. His recent history showed that one of his close relatives had died recently, and everybody was trying to explain his headache with his recent deep sorrow. He had always been a thin person with no positive medical history. He had been a heavy smoker and opium addict for a long period of time.

The patient was discharged with a tricyclic antidepressant. After a week, however, he returned with worsening of the headache and vertigo, both of which were aggravated by standing and improved in the supine position. The patient also complained of nausea and vomiting. 

Neurologic examination was not significant, except for diffuse diminished deep tendon reflexes (DTR). No sign of meningeal irritation was detected. Arterial pulses were all normal. There was no tinnitus or hearing loss. 

The patient was admitted to the local hospital to manage his severe headache by supportive care and for further investigation.

On physical examination, blood pressure, heart rate, respiratory rate and temperature were normal. Laboratory findings were as follows:

Hb=14.2 gr/dl, white blood cell count=21500/mm3, normal MCV, and MCHC and MCH. C-reactive protein (CRP) was positive. Erythrocyte sedimentation rate (ESR) was normal. Coagulation tests including prothrombin and partial thromboplastic times were unremarkable.

**Figures 1. a, b, c:**
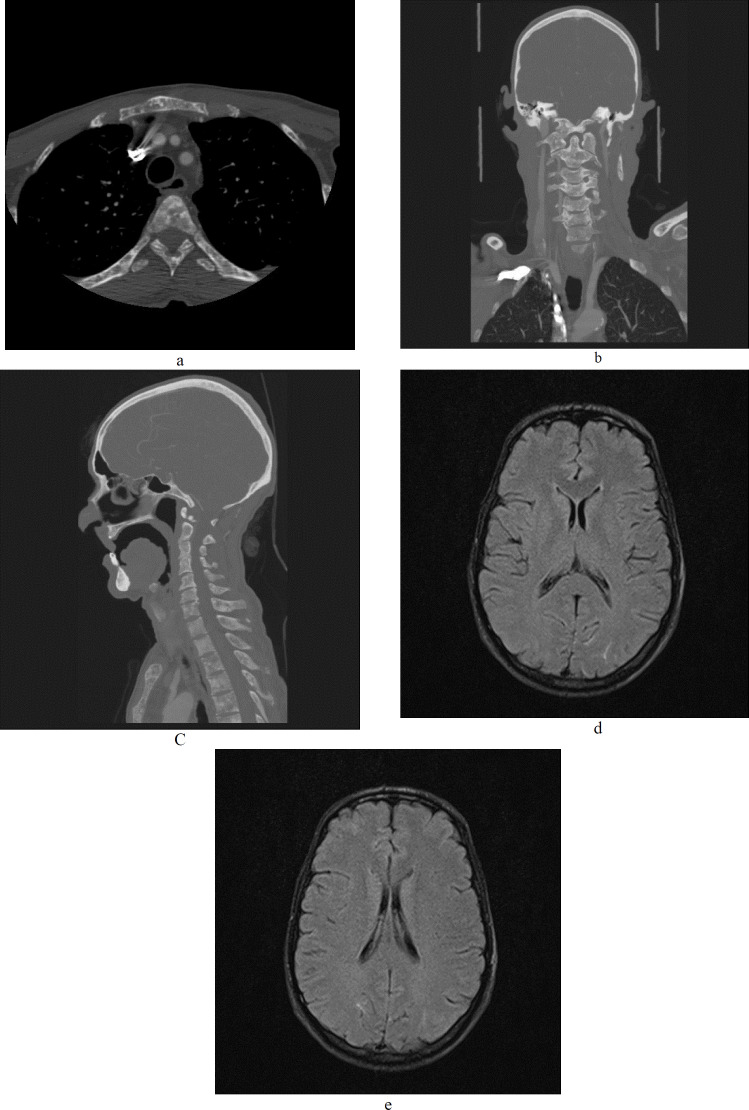
Axial, coronal, and sagittal CT scans of chest and neck showing multiple sclerotic lesions in the vertebral bodies, posterior elements, ribs, and sternum which cause a diffuse inhomogeneous appearance. **d, e:** Axial FLAIR image of brain showing increased signal intensity in the subarachnoid space and sulci of both occipital lobes

Blood urea nitrogen (BUN)=49 mg/dl, Cr=1 mg/dl, Na=138 mEq /L, and K=4 mEq /L. Calcium, phosphate and uric acid were all within normal limits. Arterial blood gas was also normal. The patient was referred to the central main hospital for imaging studies. MRI and CT scan of the brain, abdomen, and chest were performed. Axial, coronal, and sagittal CT scans of the chest and neck showed multiple sclerotic lesions in the vertebral bodies, posterior elements, ribs, and sternum which caused a diffuse inhomogeneous appearance (Figs. 1a, b, c). 

Carotid doppler sonography was also performed and was also normal. Axial FLAIR images of the brain showed increased signal intensity in the subarachnoid space and sulci of both occipital lobes (Figs. 1 d, e). 

A lumbar puncture was planned, but the patient’s condition deteriorated; he became unconscious and died after cardiorespiratory arrest.

**Figure 2:**
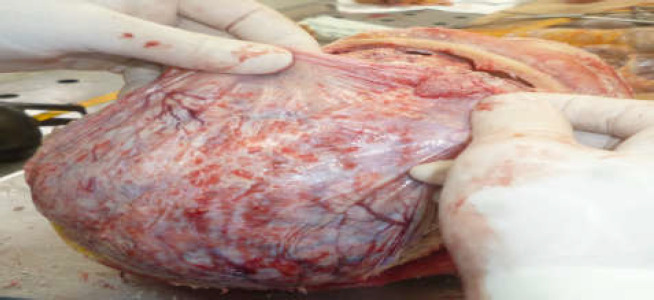
Autopsy showed multiple small excrescences on the meningeal membrane

Autopsy was performed and showed multiple small excrescences on the meningeal membrane (Fig. 2). 

**Figures 3a, b:**
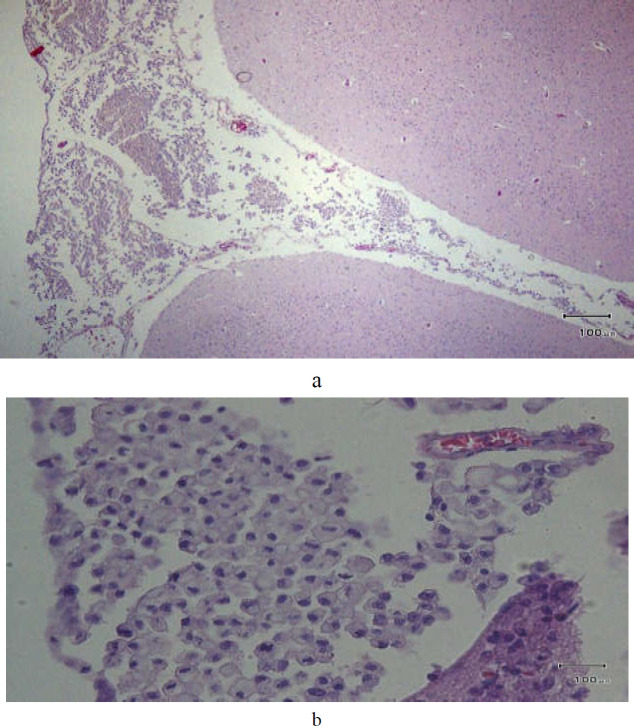
Histologic examination of the brain showed subarachnoid space filled with signet ring cells

**Figure 4:**
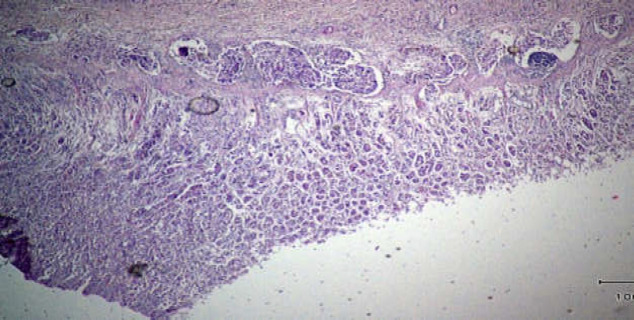
Sections from gastric necropsy showed malignant signet ring cells in the gastric wall

Histologic examination of the brain showed subarachnoid spaces filled with signet ring cells (Figs. 3a, b). Other parts of the body and all the organs were normal except for a large ulcer in the stomach, the histopathology of which was signet ring adenocarcinoma (Fig. 4).

A post-mortem study of the cerebrospinal fluid showed high protein and lactate dehydrogenase (LDH) as well as low glucose. There were also numerous signet ring cells. 

## Discussion

Primary presentation of gastric adenocarcinoma with neurological signs and symptoms of leptomeningeal carcinomatosis is an extremely rare event. There have been only 9 reported cases of primary presentation of gastric adenocarcinoma with leptomeningeal carcinomatosis in the past 20 years (4-12). Table 1 shows the characteristics of these 9 cases as well as the current case. As the table shows, the most common origin of patients has been Asia, i.e. Japan, China, Korea, and Taiwan, and the patients presented with different neurological signs and symptoms for a short duration. The most common presenting symptom in these patients as well as in our case has been headache with and without neck pain (5,7-9,11,12). Other neurological signs and symptoms have included sudden dizziness, blurred vision, diplopia, hearing and/or vision loss (6,8-11). 

The method of primary diagnosis in most of the previous cases has been lumbar puncture and CSF cytology to find the cause of neurological signs and symptoms (4-7, 11, 12). In just one case from Switzerland, the primary diagnosis was made by post-mortem CSF cytology.^12^ In the reported cases, CT scans were normal and not helpful in the primary diagnosis of leptomeningeal carcinomatosis. In the majority of these cases, however, MRI was abnormal and showed leptomeningeal contrast enhancement and ventricular dilatation (4-12). 

The whole duration of neurological symptoms in gastric cancers presenting with neurological signs and symptoms of leptomeningeal carcinomatosis from the beginning to the death of patients has not been longer than 2-4 months, and this condition has had a very poor prognosis (4-12).

In conclusion, intractable headache, dizziness, and other neurological signs without an underlying cause should be considered as a possible sign of leptomeningeal metastasis in end-stage cancers with poor prognoses.

**Tables 1:** Clinicopathologic characteristics of the 9 reported cases of gastric adenocarcinoma with primary neurologic presentation secondary to leptomeningeal carcinomatosis

Author	Year	Country	Age/ Sex	Chief Complaint	Duration of Symptoms	Primary Method of Diagnosis	Brain CT Scan	Brain MRI	Outcome
Fuchizaki et al.^4^	2005	Japan	42/M	Unsteady gate	1 month	CSF Cytology	Normal	Leptomeningeal contrast enhancement	Died after 49 days
Kon et al.^5^	2014	Japan	22/M	Headache	2 months	CSF Cytology	NR*	Cerebral edema and ventricular dilatation	Died after 2 months
Hayashi et al.^6^	2010	Japan	77/F	Bilateral blindness	1 month	CSF Cytology	NR*	Ring enhancement of surrounding optic nerve	Died after 2 weeks
Guo et al.^7^	2014	China	40/F	Headache	2 months	CSF Cytology	Cerebral edema and ventricular dilatation	Hydrocephalus and parenchymal swelling	Died after a month
Ohno et al.^8^	2010	Japan	62/M	Headache and sudden deafness	1 week	PET** and gastric wall thickening	NR*	Bilateral thickening and enhancement of both vestibulocochlear nerves	Died after 12 weeks
Lee et al.^9^	2007	Korea	49/F	Headache and dizziness	10 days	Gastric Biopsy	NR*	Normal	NR*
Ho et al.^10^	2016	Taiwan	28/F	Blurred vision	5 days	CT scan and biopsy	Normal	Leptomeningeal enhancement	Died after 2 weeks
Braeuinger et al ^11^	2005	Germany	68/M	Headache and diplopia	1 week	CSF cytology	Normal	Normal	Died after 2 months
Hollingen et al.^12^	2002	Switzerland	59/F	Headache confusion back pain	4 days	CSF cytology	New large hypodense lesions	Multiple parenchymatous lesions with strong enhancement after contrast application	Died after 11 days
Current	2019	Iran	52/M	Intractable headache	1 week	CSF cytology	Normal	Leptomeningeal enhancement	Died after a month

*NR: Not Reported;**PET: Positron emission tomography; Table 1: Clinicopathologic characteristics of the 9 reported cases of gastric adenocarcinoma with primary neurologic presentation secondary to leptomeningeal carcinomatosis

## Conflict of interests

The authors declare that they have no conflict of interest.

## References

[1] Saad N, Alsibai A, Hadid TH (2014). Carcinomatous meningitis due to gastric adenocarcinoma: A rare presentation of relapse. World J Gastrointest Oncol.

[2] Tomita H, Yasui H, Boku N, Nakasu Y, Mitsuya K, Onozawa Y (2012). Leptomeningeal carcinomatosis associated with gastric cancer. Int J Clin Oncol.

[3] Abdo AA, Coderre S, Bridges RJ (2002). Leptomeningeal carcinomatosis secondary to gastroesophageal adenocarcinoma: A case report and review of the literature. Can J Gastroenterol.

[4] Fuchizaki U, Ohta H, Kaneko S (2005). Image of the month. Gastroenterology.

[5] Kon T, Funamizu Y, Miki Y, Tomiyama M, Baba M, Kurotaki H, Wakabayashi K (2014). An autopsy case of meningeal carcinomatosis with parenchymal invasion through the cranial and spinal nerve roots. Neuropathology.

[6] Hayashi Y, Kato T, Tanaka Y, Yamada M, Koumura A, Kimura A (2010). Markedly Ring-enhanced Optic Nerves Due to Metastasis of Signet-ring Cell Gastric Carcinoma. Int Med.

[7] Guo JW, Zhang XT, Chen XS, Zhang XC, Zheng GJ, Cai YF (2014). Leptomeningeal carcinomatosis as the initial manifestation of gastric adenocarcinoma: A case report. World J Gastroenterol.

[8] Ohno T, Yokoyama Y, Aihara R, Mochiki E, Asao T, Kuwano H (2010). Sudden Bilateral Sensorineural Hearing Loss as the Presenting Symptom of Meningeal Carcinomatosis of Gastric Cancer: Report of a Case. Surg Today.

[9] Lee HG, Lee B, Kim SM, Suh BJ, Yu HJ (2007). A Case of Gastric Adenocarcinoma Presenting as Meningeal Carcinomatosis. Korean J Internal Med.

[10] Ho TH, FC Y, HW K, SJ C, Lee JT, LW W, HC C (2016). Acute lethargy in a young woman due to latent disseminated cancer mimicking bacterial meningitis: a diagnostic pitfall. Am J Emerg Med.

[11] Braeunigera S, Mwrina C, Mawrina C, Malfertheinerb P, Schildhausc HU, Seilerc C (2005). Gastric adenocarcinoma with leptomeningeal carcinomatosis as the presenting manifestation: an autopsy case report. Eur J Gastroenterol Hepatol.

[12] Hollingera P, Humma AM, Weisb J, Sturzeneggera M (2002). Meningeal Carcinomatosis: Two Unusual Clinical, Laboratory, and Radiological Presentations. Eur Neurol.

